# The Release of Immunosuppressive Factor(s) in Young Males Following Exercise

**DOI:** 10.3390/s120505586

**Published:** 2012-05-02

**Authors:** Ye Tian, Jinlei Nie, Tom K. Tong, Julien S. Baker

**Affiliations:** 1 China Institute of Sport Science, 11 Tiyuguan Road, Dongcheng District, Beijing 100061, China; 2 School of Physical Education and Sports, Macao Polytechnic Institute, Macao, China; E-Mail: jnie@ipm.edu.mo; 3 Dr. Stephen Hui Research Centre for Physical Recreation and Wellness, Department of Physical Education, Hong Kong Baptist University, Hong Kong, China; E-Mail: tongkk@hkbu.edu.hk; 4 Institute of Clinical Exercise and Health Sciences, School of Science, University of the West of Scotland, Hamilton, Scotland, UK; E-Mail: jsbaker@uws.ac.uk

**Keywords:** exercise, immunosuppressive factor, delayed onset muscle soreness

## Abstract

It has been shown that a suppressive protein, acting as an immune suppressor, is generated in animals and humans under particular stresses. However, studies related to immunosuppressive factors in response to the stress resulting from acute exercise are limited. This study compares the effects of pre- and post-exercise human serum on concanavalin A stimulated lymphocyte proliferation of mice. In the present study, blood samples in eight male undergraduates (age 21 ± 0.7 years) were taken before and immediately after ten sets of exercise consisting of 15 free and 30 10-kg loaded squat jumps in each set. The suppression of lymphocyte proliferation was analysed with high pressure liquid chromatography. It was noted from the result of gel chromatography columns that the post-exercise values of the suppression of lymphocyte proliferation, in comparison to corresponding pre-exercise values, were generally greater with significant differences observed in 7.5th–9th min post-exercise eluates (P < 0.05). Such findings suggest that intense eccentric type exercise may lead to generation of immunosuppressive factor(s) in young males.

## 1. Introduction

Stress could affect immunologic function in animals and humans. The decline of resistibility to polyomavirus and pauscher virus has been demonstrated in mice which underwent electric shocks for 6 h daily [1]. It was also noted that lymphocyte proliferation in rats in response to concanavalin A (Con A) was suppressed in the inescapable electric shocks [2]. The degree of serum immunosuppressive activity paralleled the severity of stress [3].

Some studies have shown that injury stress can result in the generation of immunosuppressive factors in blood, most of which are peptides or proteins. Constantian [4] first reported that a low molecular weight (<10,000 Daltons) peptide fraction in the serum from burn patients was capable of inhibiting the peripheral lymphocyte proliferation of normal subjects. Since then, various suppressive factor(s) in serum obtained from patients suffering from severe injury stress, such as surgical operation, trauma, hemorrhage, *etc.*, have been identified [5–7]. The generation of suppressive factor(s) is thought to be due to damaged or inflamed tissue as the more severe the tissue injury or inflammation, the stronger the inhibition of the serum on lymphocyte proliferation [5–7].

Under restraint stress in mice and rats, a suppressive protein, which acts as an immune suppressor, would be generated in peripheral lymph tissue and released into the blood stream. Blood lymphocyte proliferation induced by Con A in the stressed animals is in turn inhibited compared with their control counterparts and the molecular weight of suppressive protein is 155,000 and 370,000 Daltons [8,9]. However, little was known whether homologous suppressive peptide or protein would be generated in humans under non-injury stress demonstrated by intense exercise.

It is known that strenuous exercise induces immunosuppression. Such a response explains partly the increased risk of infection in athletes [10]. However, it was not clear whether serum immunosuppressive factor(s) would be generated after intense exercise in humans. This study was designed to examine the generation of immunosuppressive factors in response to exercise. Since the model of lymphocyte proliferation induced by Con A in mice has been well established for testing the circulating immunosuppressive polypeptide of humans in clinical situations [11], the generation of immunosuppressive factors in humans in response to exercise was revealed by comparing the effects of pre- and post-exercise human serum on the Con A-induced lymphocyte proliferation in the mice model.

## 2. Experimental Section

### Subjects

Eight healthy male adults (mean age, 21.0 ± 0.7 years; mean height, 172.8 ± 3.9 cm; mean weight, 62.4 ± 3.2 kg) volunteered to take part in the experiment. All subjects were active university students who participated in recreational activities such as jogging, football, or basketball two to three times per week. After a routine medical screening, subjects were informed of the procedures to be employed in the study and associated risks, and they provided written informed consent. This study was approved by the Ethical Committee on the Use of Human and Animal Subjects in Research at the local university.

### Experimental Design

The effects of pre- and post-exercise human serum on concanavalin A-stimulated lymphocyte proliferation of mice were examined by performing repeated bouts of eccentric exercise of squat jumps, which has been shown to be associated with the occurrence of muscle impairment, delayed muscular soreness and acute inflammation in the leg muscles [12]. Furthermore, the isokinetic strength of the leg muscles, ratings of perceived exertion (RPE) and that of perceived soreness in exercising legs (RPS) were also evaluated before and after exercise.

### Protocol

The exercise protocol was composed of ten sets of exercise sessions, with 15 free and 30 10-kg loaded squat jumps in each set. There was a 2 min break between sets. The duration of exercise was 35–45 min. All subjects were requested to perform the exercise with maximum effort. Subjects were requested to refrain from intense physical training 72 h prior to the test.

### Measurements

*RPE and RPS*-RPE (6–20) and RPS (1–12) based on the Borg Scale [13,14] were evaluated before the jumping exercise and each day for seven days after the exercise was assessed.

### Muscle Function

The Biodex Multi-joint System (Biodex Medical System, Inc, Shirley, NY, USA) was used to measure the torque around the knee joint during flexion and extension. The subjects were tested in a sitting position in a positioning chair, with the knee joint aligned with the axis of the dynamometer. Before each test, the machine was calibrated for torque, effect of gravity and range of motion (ROM) according to the general operating instructions provided by Biodex. The isokinetic strength test was performed at a speed of 60° s^−1^ in the muscle groups related to extension and flexion of the knee joint. The peak torque (PT), which was defined as the maximum torque produced by the knee at any point in the ROM, was recorded. The peak torque per unit body weight (PT/BW) was calculated in order to eliminate the impact of body weight [15,16]. PT and PT/BW were measured immediately before and after, and 24 h after the jumping exercise.

### Immunosuppressive Activity

An animal model has long been used for examining the human immune function, and is acceptable for testing circulating immunosuppressive polypeptide *in vitro* [11]. The testing procedure has been described previously [11]. For each blood sampling, venous blood (5 mL) was drawn from the antecubital vein using a venous puncture with subjects in a sitting position, and the blood was allowed to clot at room temperature. The clotted blood sample was then centrifuged at 2,000 *g* for 20 min. LACA inbred mice (6–8 weeks, male) were sacrificed by cervical dislocation followed by celiotomy and harvesting of mesenteric lymph nodes. The lymph nodes cells were obtained by gentle squeezing and washing twice with Hanks' solution. The lymphocytes were suspended in RPMI 1640 medium (GIBCO) containing 20% heat-inactivated fetal bovine serum, l-glutamine (20 mmol·L^−1^). The antibiotics (100 U·mL^−1^ penicillin, 100 μg·mL^−1^ streptomycin) were placed in wells of a 96-well flat-bottom plate with concanavalin A (Con A, Sigma, St Louis, MO, USA) and various dilutions (1:32, 1:64) of stressed or control sera. The number of lymphocytes in each well was 10^5^ cells. The final concentration of Con A was 2 μg·mL^−1^. Each dilution of the serum in culture was performed in triplicate. The cultures were incubated at 37 °C in a humidified atmosphere of 15% CO_2_. [^3^H] thymidine (Atomic Energy Research Institute of China) was added to each culture after 42 h incubation and the cells were harvested on glass fiber filters after another 6 h incubation. Radioactivity was assayed in a liquid scintillation counter to analyse the incorporation of radioactivity into DNA. The mean counts per minute were calculated from the triplicate wells. The results were expressed as counts per minute (CPM), which are the median counts per minute of the cells incubated with different sera.

### Serum Fractionation

Bio-Sil TSK 125 (300 × 7.5 cm, Bio-Rad Co.) gel filtration columns were first equilibrated with a mobile phase acetic acid buffer (50 mmol·L^−1^, pH 7.4) and then calibrated with molecular standards including thyroglobulin (MW 670,000), γ-globulin (MW 158,000), ovalbumin (MW 44,000), myoglobin (MW 17,000) and vitamin B_12_ (MW 1,350). The elution was initiated after injection of 200 μL of control or stressed serum. The elution was performed with a flow rate of 1 mL·min^−1^ at 20 °C. Proteins eluted from the gel filtration column were detected by monitoring the eluate continuously for UV absorbance at 280 nm. The eluate was collected every 0.5 min to tubes, all of which was lyophilised and redissolved in distilled water (40% in vol.) and RPMI 1640 (60% in vol.). Redissolved volume was half of that before lyophilisation. 12.5 μL of each tube was placed in wells of a 96-well flat-bottom plate with concanavalin A (2 μg·mL^−1^) and normal lymphocytes (10^5^ cells in each well) with RPMI 1640 medium. All assays were done in triplicate. The duration of incubation, the method of harvesting and the test of the suppressive activity were the same as those described above. The molecular weight was determined according to molecular standards.

### Statistical Analyses

One-way ANOVA with repeated measures was computed to examine the differences in muscle strength, RPE and RPS across time points. Paired two-tailed *t* test was computed to reveal the differences between pre-exercise and post-exercise in lymphocyte proliferation of sera. Two-way ANOVA with repeated measures was applied to examine the differences in the lymphocyte proliferation of eluate obtained from sera across pre-exercise and post-exercise. *Post-hoc* analyses using Newman-keuls were performed when the main effect was significant. All tests for statistical significance were standardised at an alpha level of P < 0.05 and all results were expressed as mean ± SD.

## 3. Results and Discussion

### 3.1. Results

**Figure 1.**
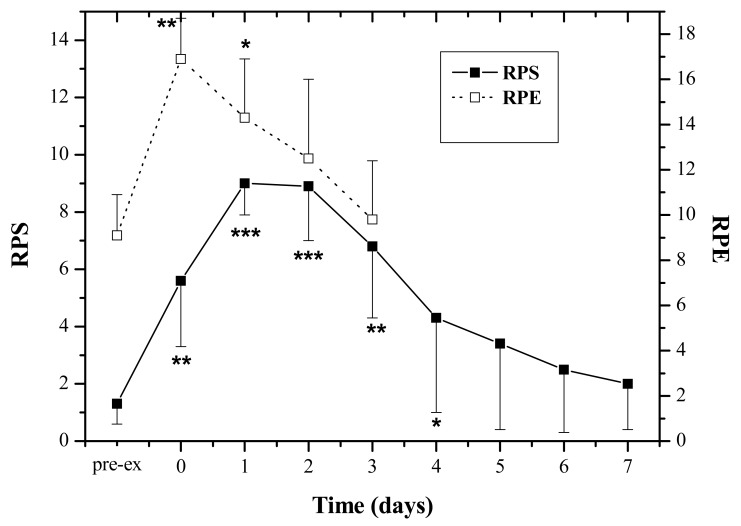
Mean ± SD of ratings of perceived exertion (RPE) and perceived soreness (RPS) of all subjects (n = 8) before (Pre) and immediately (0), one (1), two (2), three (3), four (4), five (5), six (6) and seven (7) days after exercise. * Significantly different from corresponding Pre-ex value, P < 0.05; ** P < 0.01; *** P < 0.001.

The subjects experienced moderate physical exertion during the exercise as RPE increased significantly after the exercise (Pre: 9.1 ± 1.8; Post: 14.3 ± 2.6, P < 0.01). The muscle soreness revealed by RPS peaked at 24–48 h by comparing with the pre-exercise value (P < 0.001), and attenuated 5 days post-exercise, and finally subsided seven days afterwards (Figure 1).

Isokinetic Strength—Compared with pre-exercise values, the PT and PT/BW of knee extensor significantly reduced immediately post-exercise (P < 0.05) and the declines remained for at least 24 h (P < 0.05). For the PT and PT/BW of the knee flexor, no statistical difference was found between pre- and post-exercise values (Table 1).

**Figure 2.**
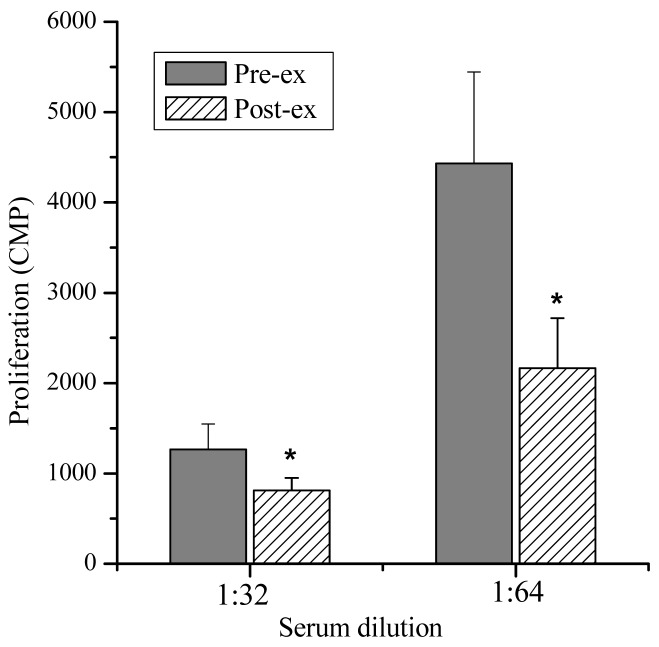
Effects of the sera obtained from subjects on normal mice lymphocyte proliferation. Data are presented as mean ± SD (n = 8). * P < 0.05, pre-exercise (Pre-ex) *vs.* post-exercise (Post-ex).

**Figure 3.**
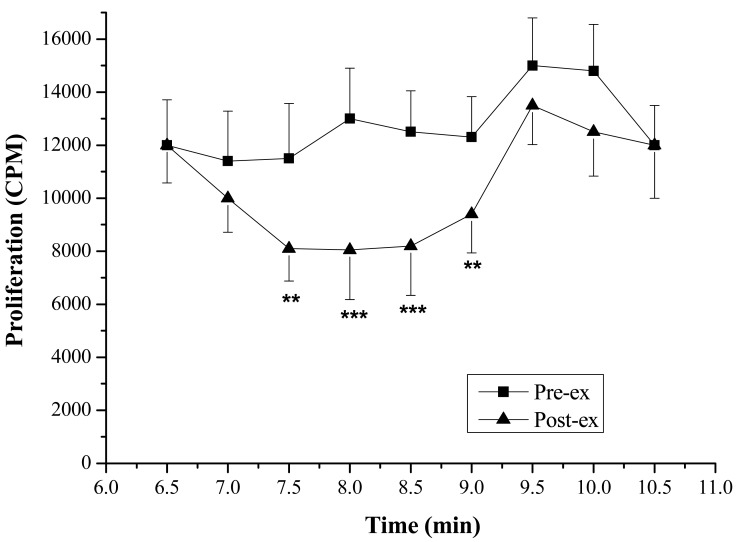
Effects of HPLC eluate obtained from sera on normal mice lymphocyte proliferation. Data are presented as mean ±SD (n = 8). ** P < 0.01, pre-exercise (Pre-ex) *vs.* post-exercise (Post-ex); *** P < 0.001.

Lymphocyte Proliferation and Molecular Weight—The lymphocyte proliferation in mice induced by Con A in post-exercise (818 ± 138, 2,164 ± 554 CPM) was significantly lower than that of pre-exercise (1,264 ± 283, 4,434 ± 1,010 CPM; P < 0.05), indicating that lymphocyte proliferation could be suppressed significantly by the exercise stress serum (Figure 2). Furthermore, marked suppressive activities were observed in the eluates of the 7.5th, 8.0th, 8.5th and 9.0th min (Figure 3). The molecular weights of these fractions, measured by calibrating the column with known molecular weight standards, were 137,000–256,000 Dalton.

### 3.2. Discussion

In the present study, acute eccentric exercise that did not cause direct tissue damage was used as a stressor to investigate the corresponding changes in the immune function of humans. The stressed serum was able to inhibit the normal lymphocyte proliferation in mice. This result was similar to that reported by Fan, who used restraint stress as a stressor [8,9]. The current findings suggested that there might be an immunosuppressive factor(s) in stressed serum that inhibits lymphocyte proliferation.

In order to determine the molecular weight of the immunosuppressive factor(s), gel filtration HPLC was used. For the sample cleanup, a Sep-Pak C18 cartridge was adopted. The eluate with suppressive activity from the Sep-Pak C18 cartridge was applied to a gel filtration HPLC column. The molecular weights of the active ingredient, estimated with molecular weight standards, were identified as 137,000–256,000 Dalton.

Although the mechanisms in the generation of the immunosuppressive factor(s) were not clear in the present study, some evidence might be found from the studies in which restraint stress was used as a stressor in mice and rats.

In a study on restraint stress, the suppressive activity was totally abolished by general anesthesia with urethane. This suggested that the central nervous system played a very important role in this process [8,9]. A study of Wang *et al.* [17] indicated that the drop in the total number of lymphocytes was not significant in the production of the serum suppressive factor(s). However, the ratio of T to B cells might be relevant to the production of the factor. Besides, inhibition of the production was observed in a nude mouse (an animal showing a lack of T cell activity), which also supported the conjecture that T cells were involved in the production of the inhibitory factor.

Other than these findings, it was reported that with the injection intracerebroventricularly of IL-1 receptor antagonist (IL-1ra) in mice, there was a significant decrease in the generation of the serum suppressive factor(s) in a dose-dependent manner. Intracerebroventricular injection of IL-1 beta enhanced the generation of the suppressive factor(s). The results revealed that IL-1 was involved in mediating the generation of suppressive factor(s) in the brain. Moreover, extracts from the lymph node and spleen in mice and rats subjected to restraint stress suppressed lymphocyte proliferation significantly, but extracts from the brain, skeletal muscle and thymus gland showed no effect on lymphocyte proliferation, which suggested that a suppressive factor for lymphocyte proliferation might be induced selectively in the lymph node and spleen. These results, taken together, indicated that a suppressive factor, under restraint stress and the control of the central nervous system, might be generated in peripheral lymph tissue and then released into the blood-stream. This factor is a strong suppressor of lymphocyte proliferation [6,18,19].

Numerous studies have shown that a bout of acute exercise induced mobilisation of all lymphocyte subpopulations to the blood. After intense exercise of long duration, the concentrations of all lymphocyte subpopulations decline, the function of NK and T cells are depressed and the local production of secretory IgA in the mucosa inhibited. Also, the neutrophils increased in response to exercise and the increase continued in the period after exercise [10]. In addition, exercise-induced immunological suppression might have detrimental effects on athletes such as contracting infections. Various epidemiological studies suggested that heavy acute or chronic exercise is usually associated with an increased risk of upper respiratory tract infections [20].

Polyclonal mitogens are used in most studies on lymphocyte proliferation. This kind of mitogen can induce many or all lymphocytes of a given type to proliferate. Studies in humans showed a decline in the lymphocyte responses to the T-cell mitogens phytohemagglutinin (PHA) and concanavalin A (ConA) both during exercise and up to several hours post exercise [21]. MacNeil studied the effects of intensity and duration of exercise on lymphocyte proliferation as a measure of immunologic function in men of defined fitness. Blood samples were obtained at various times before and after exercise sessions. Lymphocyte responses to the T cell mitogen Con A were determined at each sample time through the incorporation of radiolabeled [^3^H] thymidine. Despite the differences in resting levels of [^3^H] thymidine uptake, a consistent depression in mitogenesis was present 2 h after an exercise bout in all fitness groups. All the data suggested that single sessions of submaximal exercise transiently reduced lymphocyte function in men and that this effect occurred irrespective of the subjects' fitness level [22]. It is believed that the suppression of lymphocyte proliferation may be attributed to the generation of the immunosuppressive factor(s).

A recent study found that lymphocyte proliferation was significantly suppressed by the extraction of the human tonsil. The suppression induced by the extract was partially reversed by the monoclonal antibody against immunosuppressive factor(s). In an ELISA test, the extract was able to bind to the monoclonal antibody. By immunohistochemistry, many immunosuppressive factors positive cells were found in the tonsil and lymph nodes of humans. All these results pointed to the possible existence of a protein similar to immunosuppressive factor in human peripheral lymphoid tissue [23].

The current study has some major limitations, which nevertheless provide guidance for further research. This is a small initial study with only a limited method of identifying the detailed characteristics of the immune suppressive protein observed. Because of its large molecular weight, it was not possible for the suppressive factor(s) to be obtained in our study such as those of the well-known hormones, interleukins, interferons, or the serum suppressive factor(s) induced by burn, trauma, or hemorrhage [3,7,24]. Neither were they identical with the serum suppressive factor(s) induced by restraint stress in mice and rats. They are supposed to be either new proteins induced by exercise stress or suppressive factor (s) present in normal serum, which increased significantly during exercise stress. However, at present, we still cannot exclude the possibility that the compounds might be polymers of smaller known proteins, small proteins or peptides bound to carrier molecules, or preproteins that are cleaved into active forms at the target site. Future research may wish to employ a more delicate analysis such as 2-D gel electrophoresis to solve the issue. Moreover, although the animal model used in the present study to test the relationship between human serum fraction and immune function has been accepted as a test of circulating immunosuppressive polypeptide-containing serum fraction *in vitro*, the confounding variables of using two different species (humans and mice) in the same experiment existed. Future work should determine whether treating cultured human cells with human serum would result in similar results. Finally, it would be interesting to explore further and compare the findings observed in this study using an animal exercise model. This proposal may advance further our understanding of the biochemical mechanisms and interaction responsible for the release of immunosuppressive factor.

## 4. Conclusions/Outlook

In summary, a suppressive factor(s) was generated in human serum under exercise stress. It acted as a very strong suppressor of lymphocyte proliferation induced by ConA in mice. The immunosuppressive factor(s) was proteinaceous in nature and very large in molecular weight. The factor(s) might be a new regulatory protein performing the function of a mediator between the nervous and immune system. The current findings may provide guidance for investigating exercise-induced immunological responses and adaptations, which may lead to a better understanding of the interactions between exercise stress and the immune system.

## Figures and Tables

**Table 1.** Mean ± SD of peak torque (PT, ft-lb) and peak torque/body weight (PT/BW, ft-lb/lb) of knee extensor and knee flexor at 60° s^−1^ of all subjects (n = 8) before (Pre-ex) and immediately (0 h), twenty-four (24 h) hours after exercise are shown.

	**Knee extensor**		**Knee flexor**	
	
	**PT**	**PT/BW**	**PT**	**PT/BW**
Pre-ex	160.1 ± 20.6	1.17 ± 0.12	75.9 ± 11.0	0.51 ± 0.07
0 h	128.3 ± 16.6 *	0.95 ± 0.11 *	77.6 ± 13.3	0.54 ± 0.09
24 h	123.2 ± 22.7 *	0.90 ± 0.14 *	74.0 ± 10.8	0.51 ± 0.08

* Significantly different from corresponding Pre-ex value, P < 0.05.
